# Survival benefit and immune-related toxicities after hematopoietic stem cell transplantation in Krabbe disease: a systematic review and meta-analysis

**DOI:** 10.3389/fimmu.2026.1924827

**Published:** 2026-08-13

**Authors:** Jie Zhang, Quanzhen Tan, Weiyi Zhu, Huan Yi, Ye Wu

**Affiliations:** Children’s Medical Center, Department of Pediatric Neurology, Peking University First Hospital, Beijing, China

**Keywords:** graft-versus-host disease, hematopoietic stem cell transplantation, immune-related toxicity, Krabbe disease, leukodystrophy, meta-analysis, transplant-related mortality

## Abstract

**Background:**

Krabbe disease is a rare, rapidly progressive leukodystrophy with high early mortality. Hematopoietic stem cell transplantation (HSCT) is the main disease-modifying intervention used in clinical practice and may act through donor-derived myeloid and immune-cell replacement, enzymatic cross-correction, and immunomodulation. However, its benefits and immune-related toxicities remain incompletely quantified.

**Methods:**

We conducted a systematic review and meta-analysis of studies reporting HSCT outcomes in genetically or enzymatically confirmed Krabbe disease. The primary outcome was overall survival (OS). Secondary outcomes included 5-year OS, transplant-related mortality (TRM), acute and chronic graft-versus-host disease (aGVHD and cGVHD), neurological stability, and MRI stability. For OS, publication bias or small-study effects were assessed using funnel-plot inspection and Egger’s regression test. Exploratory univariable meta-regression examined total study sample size, publication year, and Newcastle–Ottawa Scale (NOS) score, and robustness was assessed using leave-one-out sensitivity analysis.

**Results:**

Fifteen studies involving 141 patients were included. Pooled OS after HSCT was 84% (95% CI, 75%–93%), 5-year OS was 80% (69%–92%), and TRM was 7% (1%–13%). The pooled incidences of aGVHD and cGVHD were 53% (16%–90%) and 25% (0%–53%), respectively. Neurological stability was reported in only four studies including 15 patients (77%; 31%–100%), and MRI stability in four studies including 32 patients (49%; 7%–90%); both estimates showed substantial heterogeneity and wide confidence intervals. Subgroup analyses by disease-onset age, pre-transplant symptom status, and age at HSCT showed no statistically significant OS differences. Egger’s test detected no significant funnel-plot asymmetry (P = 0.569). Leave-one-out estimates ranged from 82% to 86%. Meta-regression showed inverse associations of reported OS with total study sample size and NOS score (both P < 0.001), whereas publication year was not significant (P = 0.118).

**Conclusion:**

HSCT is associated with favorable survival and relatively low pooled TRM in selected patients with Krabbe disease. However, survival should not be interpreted as preservation of neurological function, because neurological, MRI, and GVHD outcomes were reported in few studies and patients and remained heterogeneous and imprecisely estimated. These findings require cautious interpretation but provide clinically useful benchmark data for counseling and comparison with emerging cellular and gene-based therapies.

**Systematic review registration:**

https://www.crd.york.ac.uk/PROSPERO/, identifier CRD420261285624.

## 1 Introduction

Krabbe disease (globoid cell leukodystrophy, OMIM #245200) is a rare autosomal recessive lysosomal disorder caused by deficiency of galactocerebrosidase (GALC), leading to the accumulation of psychosine and progressive demyelination of both the central and peripheral nervous systems (1, 2). The disease exhibits a broad phenotypic spectrum, ranging from neonatal to adult onset. Infantile-onset Krabbe disease (IKD), usually defined by onset at or before 12 months of age, is characterized by rapid neurological deterioration and death typically before 2 years of age, whereas later-onset disease (LKD) follows a more variable and often slower clinical course (2). Although rare, with an estimated incidence ranging from 1 in 100, 000 to 1 in 310, 000 live births, Krabbe disease causes substantial neurological disability and early mortality (3–5).

Early diagnosis has improved with enzyme assays, genetic testing, and expanded newborn screening (6, 7). At present, there is no curative treatment that fully reverses neurological injury in Krabbe disease. Hematopoietic stem cell transplantation (HSCT) has been used as a disease-modifying therapy in selected patients, particularly when performed at a very early stage (6–8). From an immunological and cellular therapy perspective, the therapeutic rationale for HSCT in Krabbe disease is thought to be based largely on donor-derived hematopoietic cells that provide functional galactocerebrosidase (GALC) through cross-correction, thereby supporting metabolic correction and myelin preservation. Beyond enzyme replacement, experimental studies suggest that GALC-expressing macrophages and bone marrow transplantation may also influence macrophage-mediated, inflammatory, and neuroimmune pathways, which may further contribute to the disease-modifying effects of HSCT (9–11).

Despite these potential benefits, the clinical evidence supporting HSCT remains limited. Most available studies are small, retrospective, and heterogeneous, making it difficult to define optimal patient selection, referral timing, transplant protocols, and expected neurological trajectories. In addition, HSCT is accompanied by immune-related and transplant-related risks, including graft-versus-host disease (GVHD), transplant-related mortality (TRM), infection, and conditioning-related toxicity. These risks are particularly relevant because Krabbe disease is a non-malignant indication and many candidates are presymptomatic or very young infants.

Recent advances in cellular and gene-based therapies have raised new expectations for Krabbe disease. Preclinical studies suggest that viral vector-mediated gene delivery can correct metabolic abnormalities, slow demyelination, and extend survival (12–14). As these approaches move closer to clinical translation, well-synthesized HSCT outcome data are needed to guide risk-benefit counseling and to provide benchmark estimates for survival, immune-related toxicity, neurological outcomes, and future trial design.

Accordingly, we conducted a systematic review and meta-analysis of published clinical studies of HSCT in Krabbe disease, integrating survival outcomes, neurological and radiological outcomes, and major transplant-related immune toxicities.

## 2 Materials and methods

### 2.1 Study design and registration

This systematic review and meta-analysis was conducted in accordance with the Preferred Reporting Items for Systematic Reviews and Meta-Analyses (PRISMA) 2020 guidelines and was prospectively registered in PROSPERO (CRD420261285624). The PICOS (participants, interventions, comparators, outcomes, and study design) criteria are summarized in **Table 1**.

**Table 1:** PICOS criteria for inclusion of studies.

Parameter	Criteria
Population	Patients with a definite genetic or enzymatic diagnosis of Krabbe disease
Intervention/exposures	HSCT, regardless of donor type, stem cell source, or conditioning regimen
Comparator	No restriction on whether a control group was included
Outcome	Overall survival after HSCT; secondary outcomes included 5-year OS, TRM, aGVHD, cGVHD, MRI stability, and neurological stability
Study design	Randomized controlled trials, nonrandomized controlled trials, case-control studies, cohort studies, and case series (≥2 cases); case reports were excluded

aGVHD, acute graft-versus-host disease; cGVHD, chronic graft-versus-host disease; HSCT, hematopoietic stem cell transplantation; MRI, magnetic resonance imaging; OS, overall survival; TRM, transplant-related mortality.

### 2.2 Literature search

A comprehensive literature search was conducted across PubMed, Embase, Web of Science, the Cochrane Library, WanFang Data, China National Knowledge Infrastructure (CNKI), and VIP Information/Chinese Scientific Journals Database (VIP) from inception to January 4, 2026. Searches combined Medical Subject Headings and free-text terms related to Krabbe disease, globoid cell leukodystrophy, hematopoietic stem cell transplantation, hematopoietic cell transplantation, and bone marrow transplantation. The complete search strategy is provided in **Supplementary Figure 1**.

### 2.3 Eligibility criteria

Studies were eligible if they met the following criteria: (1) randomized controlled trials, nonrandomized controlled trials, case-control studies, cohort studies, or case series with at least two cases; (2) inclusion of adults or children with a definite genetic or enzymatic diagnosis of Krabbe disease; and (3) receipt of HSCT, regardless of donor type, stem cell source, conditioning regimen, or GVHD prophylaxis. Studies were excluded if they were reviews, animal or *in vitro* studies, single-case reports, conference abstracts without extractable data, duplicate reports without new outcome data, or studies lacking clear clinical outcomes.

### 2.4 Outcomes

The primary outcome was overall survival (OS), defined as the proportion of patients alive at the last follow-up after HSCT. Secondary outcomes included 5-year OS, MRI stability, neurological function stability, TRM, aGVHD, and cGVHD. MRI stability was defined as absence of progressive deterioration on brain MRI after HSCT at last follow-up. Neurological function stability was defined as absence of progressive deterioration of neurological function after HSCT at last follow-up. TRM was defined as death due to HSCT-related adverse events rather than disease progression. aGVHD and cGVHD were defined according to the reporting of the original studies, with aGVHD generally occurring within 100 days after HSCT and cGVHD occurring later.

### 2.5 Study selection and data extraction

Two reviewers (JZ and QT) independently screened titles, abstracts, and full texts, and extracted data using a standardized form. Extracted information was cross-checked for accuracy and consistency. A third reviewer (YW) independently reviewed the included articles to confirm data interpretation. Disagreements were resolved by discussion and consensus.

For each included study, extracted variables included study characteristics, sample size, age at onset, clinical phenotype, pre-transplant symptom status, donor type, stem cell source, conditioning regimen, GVHD prophylaxis, age at transplantation, engraftment, follow-up duration, survival, GVHD, TRM, neurological outcomes, and MRI outcomes when available. When multiple reports appeared to originate from the same institution or cohort, we reviewed study periods, patient characteristics, transplant timing, and outcome descriptions to minimize potential patient overlap. When overlap could not be excluded, the most comprehensive or most recent dataset was prioritized for the relevant outcome.

### 2.6 Risk of bias assessment

Two reviewers (JZ and QT) independently assessed the methodological quality of the included studies. Because no randomized controlled trials were included, risk of bias was assessed using the Newcastle-Ottawa Scale (NOS), which evaluates selection, comparability, and outcome/exposure domains, with a maximum score of 9. Disagreements were resolved through discussion or consultation with a third investigator (YW) (15).

### 2.7 Statistical analysis

Statistical analyses were performed using R/RStudio with the meta and metafor packages. Pooled proportions and corresponding 95% confidence intervals (CIs) were calculated for OS, 5-year OS, MRI stability, neurological stability, TRM, aGVHD, and cGVHD. Random-effects estimates were prioritized when clinical or statistical heterogeneity was present. Statistical heterogeneity was assessed using Cochran’s Q test and the I² statistic. Subgroup analyses were performed according to disease onset age, pre-transplant symptom status, and age at HSCT when data permitted. For the primary OS outcome, potential publication bias and small-study effects were assessed by visual inspection of the funnel plot and Egger’s regression test.

To explore potential sources of heterogeneity, separate exploratory univariable mixed-effects meta-regression models were fitted using total study sample size, publication year, and NOS score as study-level moderators. These variables were available for all 15 studies. A multivariable meta-regression model was not performed because of the limited number of studies and the correlation between total sample size and NOS score, which increased the risk of model overfitting and unstable estimates. Robustness of the pooled OS estimate was assessed using leave-one-out sensitivity analysis under the random-effects model. A two-sided P value <0.05 was considered statistically significant.

## 3 Results

### 3.1 Search results and study characteristics

**Figure 1:**
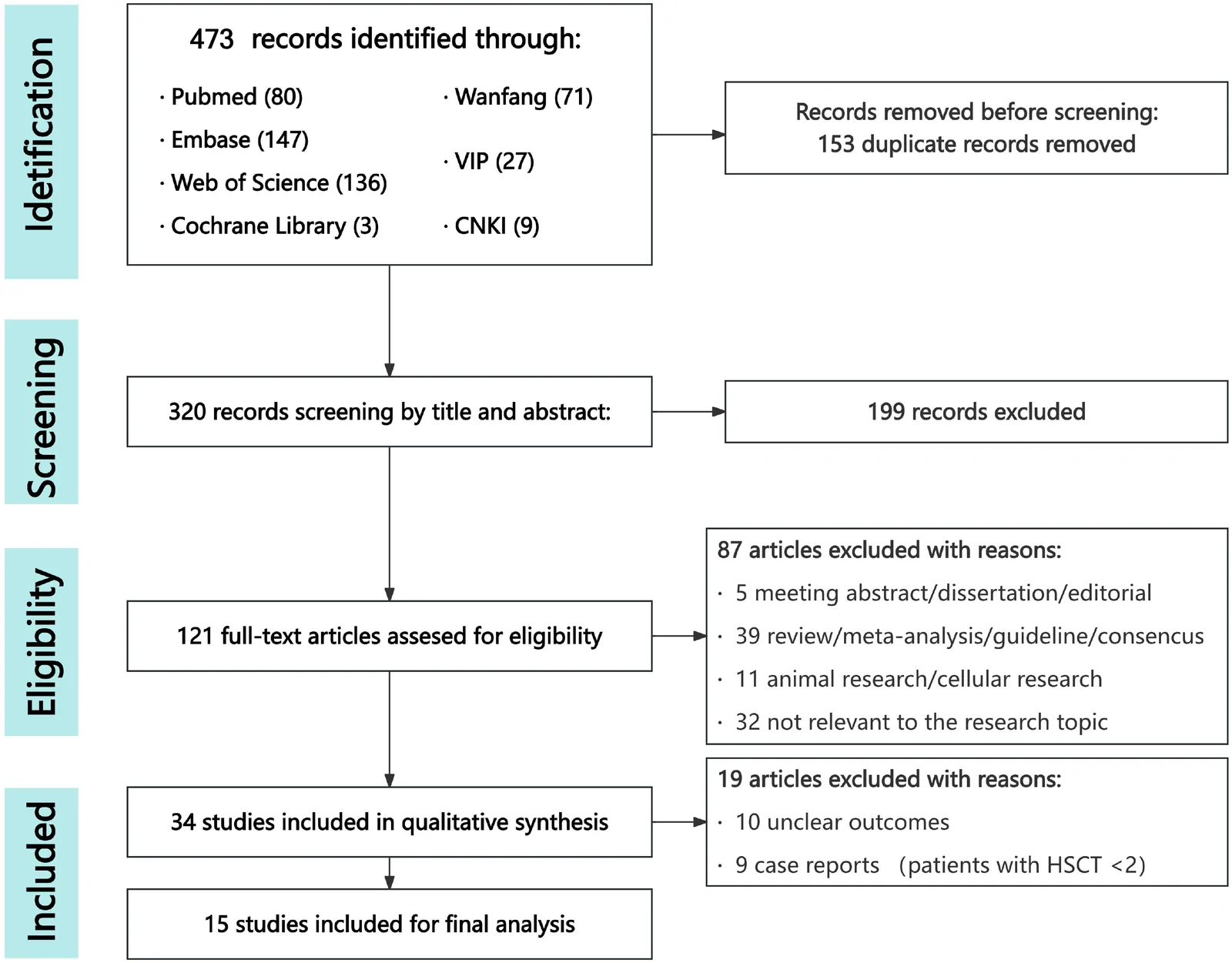
PRISMA flow diagram showing the study selection process.

The search process is summarized in **Figure 1**. A total of 473 potentially relevant publications were identified. After screening and eligibility assessment, 15 studies comprising 141 patients were included in the final analysis (7, 8, 16–28). The included studies consisted of 3 prospective cohort studies (17, 23, 24), 9 retrospective cohort studies (7, 8, 16, 18, 20, 21, 26–28), 1 case-control study (19), and 2 case series (22, 25). The main clinical and transplant characteristics are summarized in **Table 2**.

**Table 2:** Clinical and transplant characteristics of included studies.

Study	Study design	Patients included, n	Age at onset/clinical status	HSCT group	Comparator	Mean age at HSCT, years	Mean follow-up after HSCT, years	NOS score
Page, K. M. 2022 (7)	Retrospective cohort	6	Asymptomatic	HSCT	NR	0.099	0.583	3
Yoon, I. C. 2021 (17)	Prospective cohort	19	6-36m	HSCT	NR	NR	NR	5
Allewelt, H. 2018 (18)	Retrospective cohort	19	≤2m	HSCT	NR	0.074	11.2	3
Langan, T. J. 2016 (19)	Case-control study	16	7 cases: ≤6m-3y 9 cases: adolescent/adult onset	HSCT	Without HSCT	NR	NR	5
Duffner, P. K. 2012 (20)	Retrospective cohort	9	2 cases: ≤6m 5 cases: 6m-3y 2 cases: adolescent/adult onset	HSCT	NR	NR	NR	2
McGraw, P. 2005 (21)	Retrospective cohort	4	≤1y	HSCT	NR	0.542	NR	2
Gnanakumar, A. 2025 (22)	Case series	2	10d, 12d	HSCT	NR	0.081	0.284	2
Corti, P. 2005 (23)	Prospective cohort	3	≥1y	HSCT	NR	7.167	3.600	2
Wasserstein, M. P. 2016 (24)	Prospective cohort	4	≤6m	HSCT	NR	0.088	3.542	5
Gaipa, G. 2003 (16)	Retrospective cohort	3	NR	HSCT	NR	NR	NR	2
Krivit, W. 1998 (8)	Retrospective cohort	5	5y, 4y, 1m, 2 cases Asymptomatic	HSCT	NR	5.640	5.170	3
Almudhry, M. 2025 (25)	Case series	2	8y, 21y	HSCT	NR	33.000	5.000	2
Ghabash, G. 2021 (26)	Retrospective cohort	29	NR	HSCT	NR	NR	0.890	6
Wright, M. D. 2017 (27)	Retrospective cohort	18	≤7w (Asymptomatic/symptomatic)	HSCT	NR	4.089	9.500	6
Tang Xiangfeng. 2025 (28)	Retrospective cohort	2	NR	HSCT	NR	NR	NR	3

HSCT, hematopoietic stem cell transplantation; NOS, Newcastle-Ottawa Scale; NR, not reported; d, day; m, month; y, year.

The NOS scores ranged from 2 to 6, indicating overall low to moderate methodological quality. No randomized controlled trial was identified; although three studies were prospective, most evidence came from small non-randomized cohorts, a case-control study, or case series. Transplant-specific variables such as donor type, stem cell source, conditioning regimen, GVHD prophylaxis, and engraftment were not reported consistently enough to support robust quantitative subgroup analyses.

### 3.2 Overall survival after HSCT

**Figure 2:**
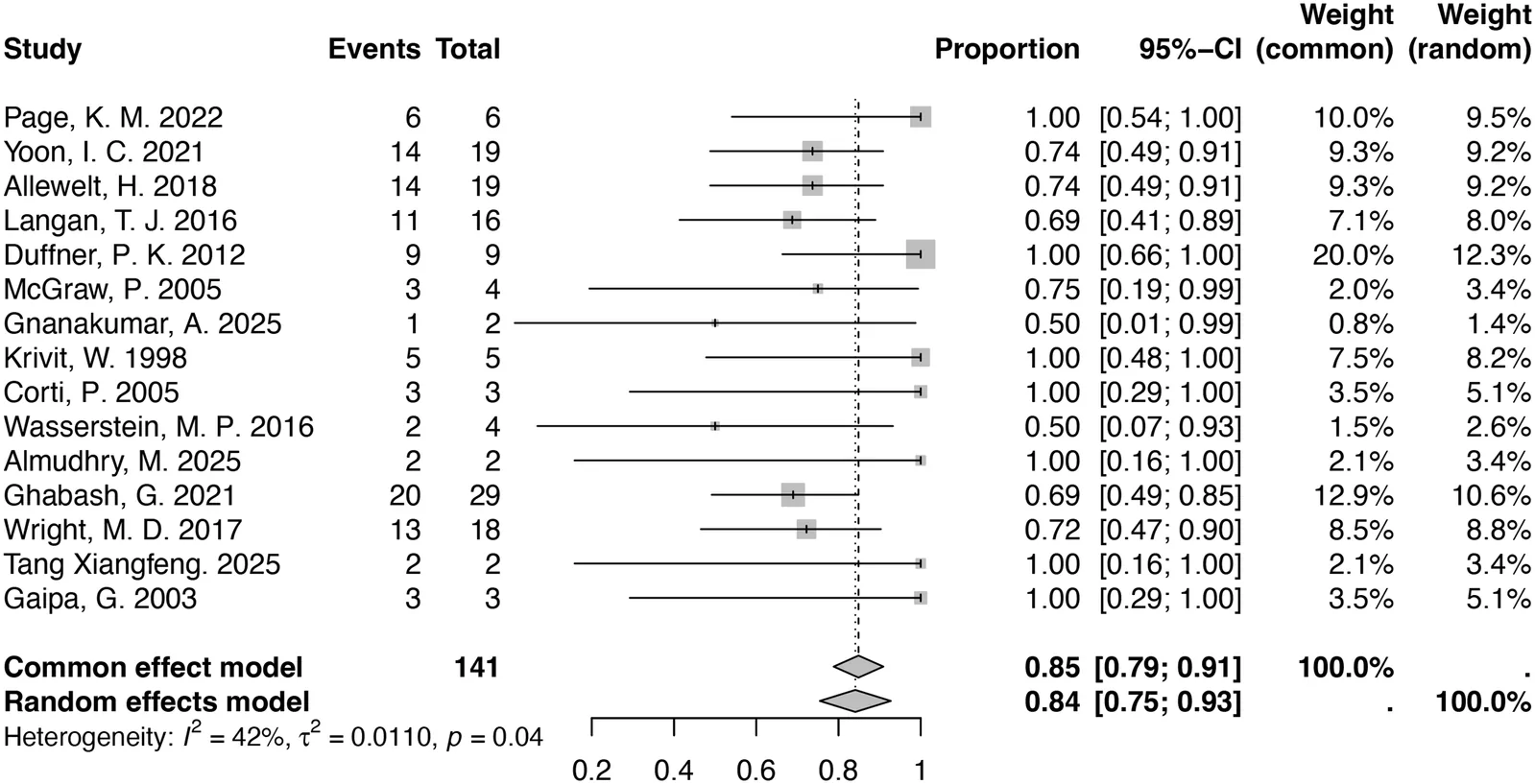
Overall survival (OS) after hematopoietic stem cell transplantation in Krabbe disease.

All 15 studies (n = 141) reported OS after HSCT. The pooled OS rate was 84% (95% CI, 75%-93%; I^2^ = 42%; P = 0.04) under the random-effects model (**Figure 2**). Data on age at HSCT were available from nine studies (7, 8, 18, 21–25, 27), with a pooled mean age at HSCT of 3.17 years (95% CI, 0.20-6.14 years; I^2^ = 98%; P < 0.01) (**Supplementary Figure 2A**). Follow-up time after HSCT was reported in nine studies (7, 8, 18, 22–27), with a pooled mean follow-up of 4.36 years (95% CI, 1.78-6.93 years; I^2^ = 98%; P < 0.01) (**Supplementary Figure 2B**).

Quantitative meta-analysis of age at disease onset and disease duration at transplantation was not feasible because these variables were incompletely reported, particularly in studies involving newborn screening cohorts. Only two studies explicitly reported these variables. In one study (8), three of five patients had reported ages at disease onset (5 years, 4 years, and 1 month) with corresponding disease durations at HSCT of 6.3 years, 2.1 years, and 1 month. Another study (22) reported two patients with disease onset at 8 years and 24 years and disease durations at HSCT of 32 years and 2 years, respectively.

### 3.3 Efficacy-related secondary outcomes

**Figure 3:**
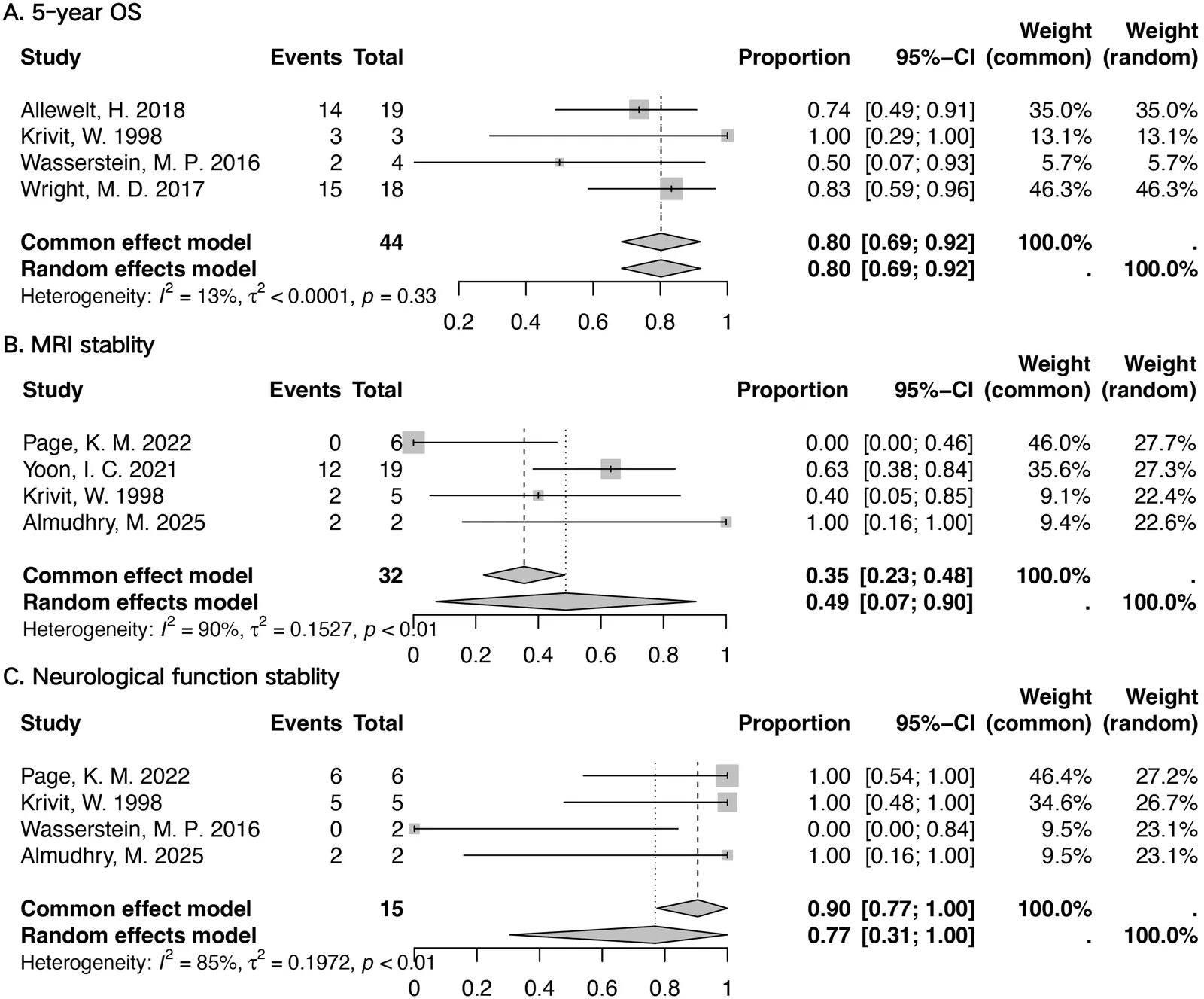
Efficacy-related secondary outcomes after HSCT. **(A)** Rate of five-year overall survival. **(B)** Rate of MRI stability. **(C)** Rate of neurological function stability.

Four studies including 44 patients reported 5-year OS (8, 18, 24, 27). The pooled 5-year OS rate was 80% (95% CI, 69%-92%; I^2^ = 13%; P = 0.33) (**Figure 3A**). MRI stability was reported in only four studies comprising 32 patients (7, 8, 17, 25), with a pooled MRI stability rate of 49% (95% CI, 7%-90%; I^2^ = 90%; P < 0.01) (**Figure 3B**). Neurological function stability was assessed in only four studies including 15 patients (7, 8, 24, 25), with a pooled rate of 77% (95% CI, 31%-100%; I^2^ = 85%; P < 0.01) (**Figure 3C**). These efficacy-related outcomes demonstrated considerable heterogeneity, particularly for MRI and neurological stability. Compared with survival, neurological and imaging outcomes were therefore much less frequently reported. Given the small numbers of contributing studies and patients, wide CIs, heterogeneous definitions, and substantial heterogeneity, these pooled estimates should be interpreted as sparse and exploratory evidence rather than definitive evidence of neurological or radiological benefit.

### 3.4 Immune-related and transplant-related safety outcomes

**Figure 4:**
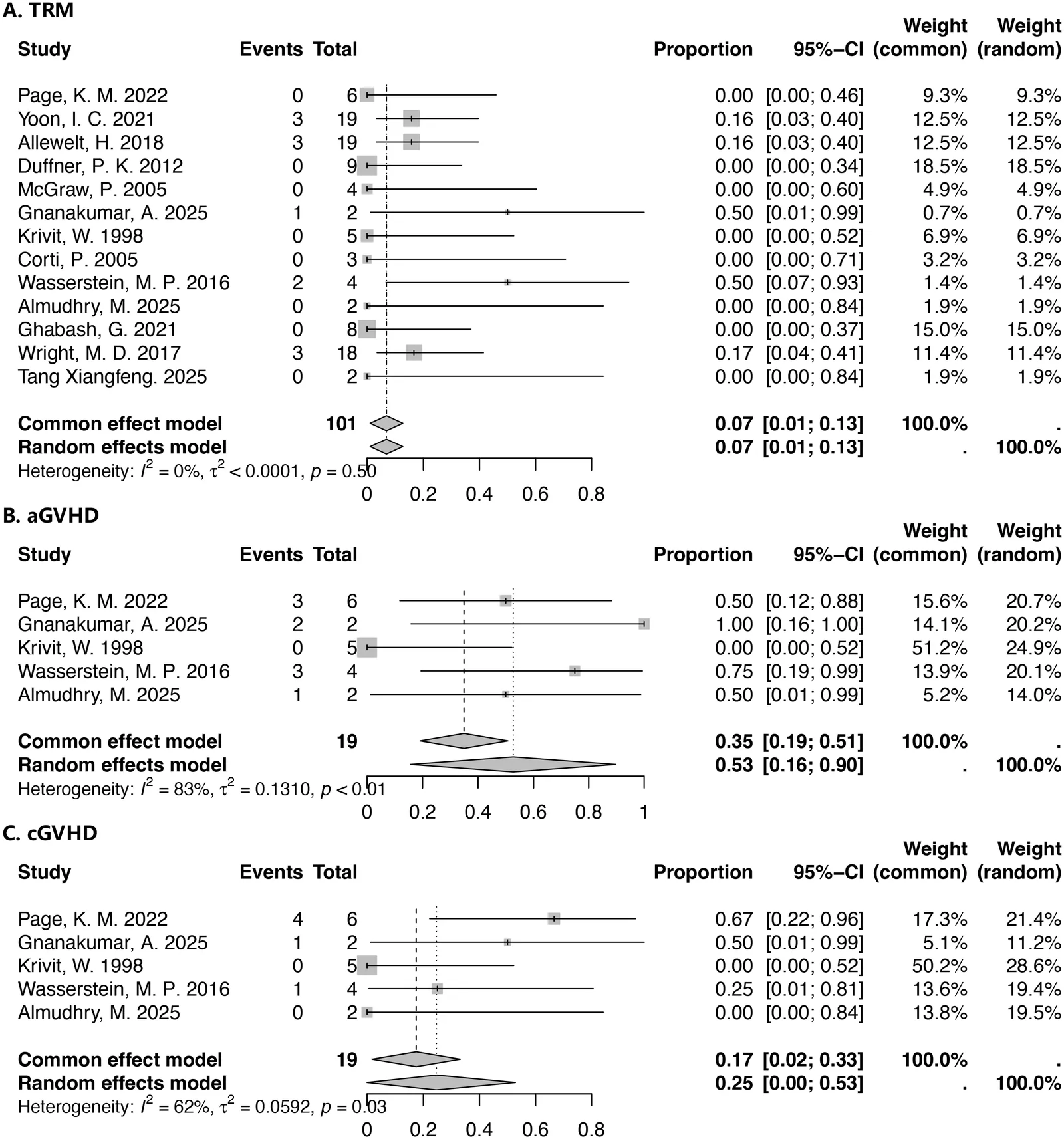
Immune-related and transplant-related safety outcomes after HSCT. **(A)** Rate of transplant-related mortality (TRM). **(B)** Rate of acute graft-versus-host disease (aGVHD). **(C)** Rate of chronic graft-versus-host disease (cGVHD).

TRM was reported in 13 studies involving 101 patients (7, 8, 17, 18, 20–28), with a pooled incidence of 7% (95% CI, 1%-13%; I^2^ = 0%; P = 0.50) (**Figure 4A**). aGVHD was reported in five studies comprising 19 patients (7, 8, 22, 24, 25), with a pooled incidence of 53% (95% CI, 16%-90%; I^2^ = 83%; P < 0.01) (**Figure 4B**). cGVHD was reported in five studies including 19 patients (7, 8, 22, 24, 25), with a pooled incidence of 25% (95% CI, 0%-53%; I^2^ = 62%; P = 0.03) (**Figure 4C**). Because both GVHD estimates were based on few patients, had wide CIs, and showed substantial heterogeneity, they should be interpreted cautiously and may not be generalizable across donor types, stem cell sources, conditioning regimens, or GVHD prophylaxis strategies.

### 3.5 Subgroup analyses

Subgroup analyses were performed to examine the effects of disease onset age (infantile-onset versus later-onset), pre-transplant symptom status (asymptomatic versus symptomatic), and age at HSCT (neonatal versus post-neonatal) on OS.

**Figure 5:**
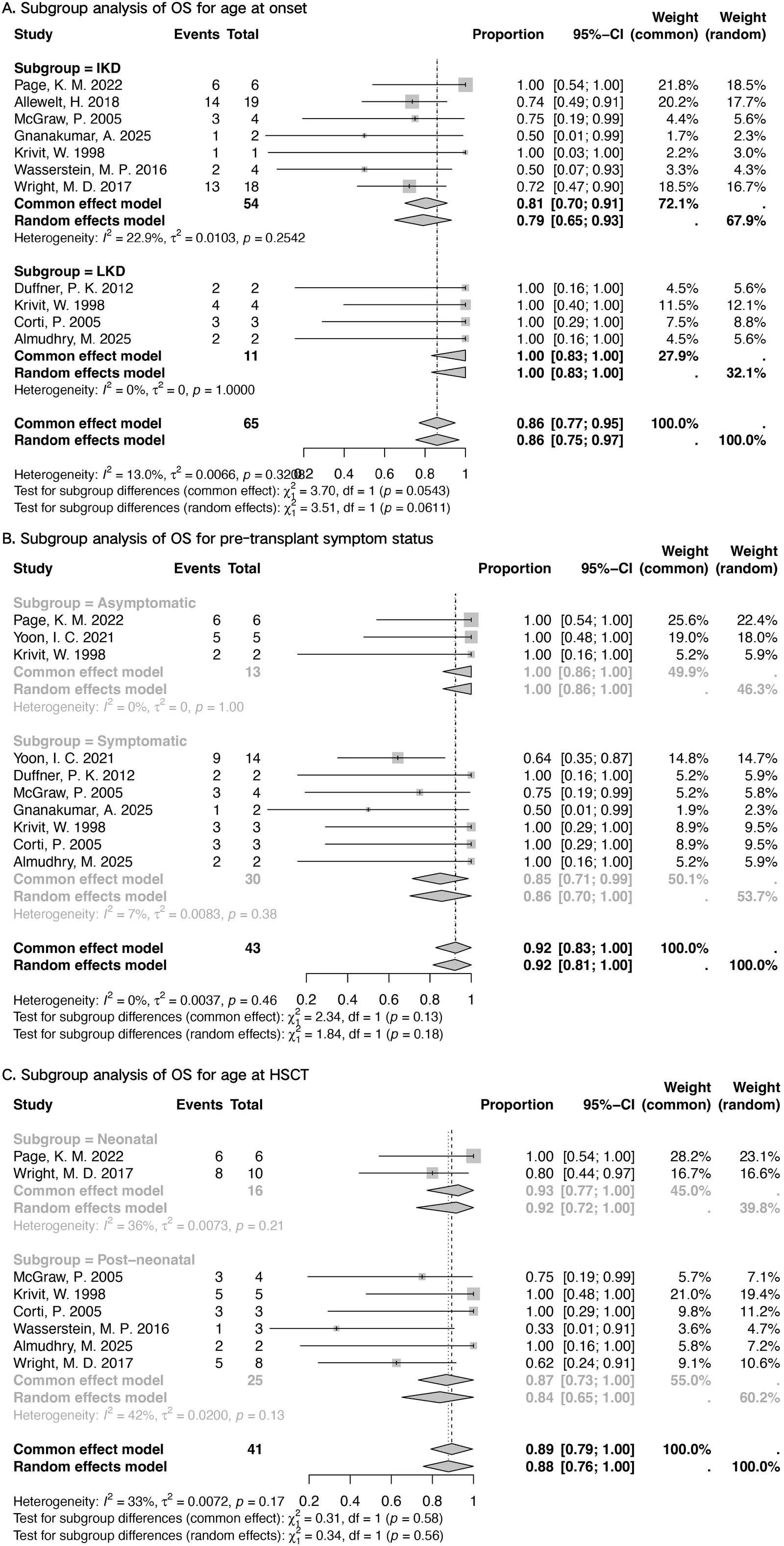
Subgroup analyses of OS after HSCT. **(A)** Subgroup analysis by age at disease onset. **(B)** Subgroup analysis by pre-transplant symptom status. **(C)** Subgroup analysis by age at HSCT.

Ten studies including 65 patients (54 infantile-onset and 11 later-onset) were available for subgroup analysis by disease onset age (7, 8, 18, 20–25, 27), showing no statistically significant difference between infantile-onset and later-onset groups (79% versus 100%; P = 0.06). Eight studies comprising 43 patients (13 asymptomatic and 30 symptomatic) reported OS according to pre-transplant symptom status (7, 8, 17, 20–23, 25), with no significant difference between asymptomatic and symptomatic patients (100% versus 86%; P = 0.18). Seven studies including 41 patients (16 neonatal and 25 post-neonatal HSCT) assessed OS by age at HSCT (7, 8, 21, 23–25, 27), and OS did not differ significantly between neonatal and post-neonatal transplantation groups (92% versus 84%; P = 0.56) (**Figures 5A–C**). Subgroup analyses according to donor type, stem cell source, conditioning regimen, or GVHD prophylaxis could not be performed because these variables were inconsistently reported.

### 3.6 Publication bias, meta-regression, and sensitivity analysis

**Figure 6:**
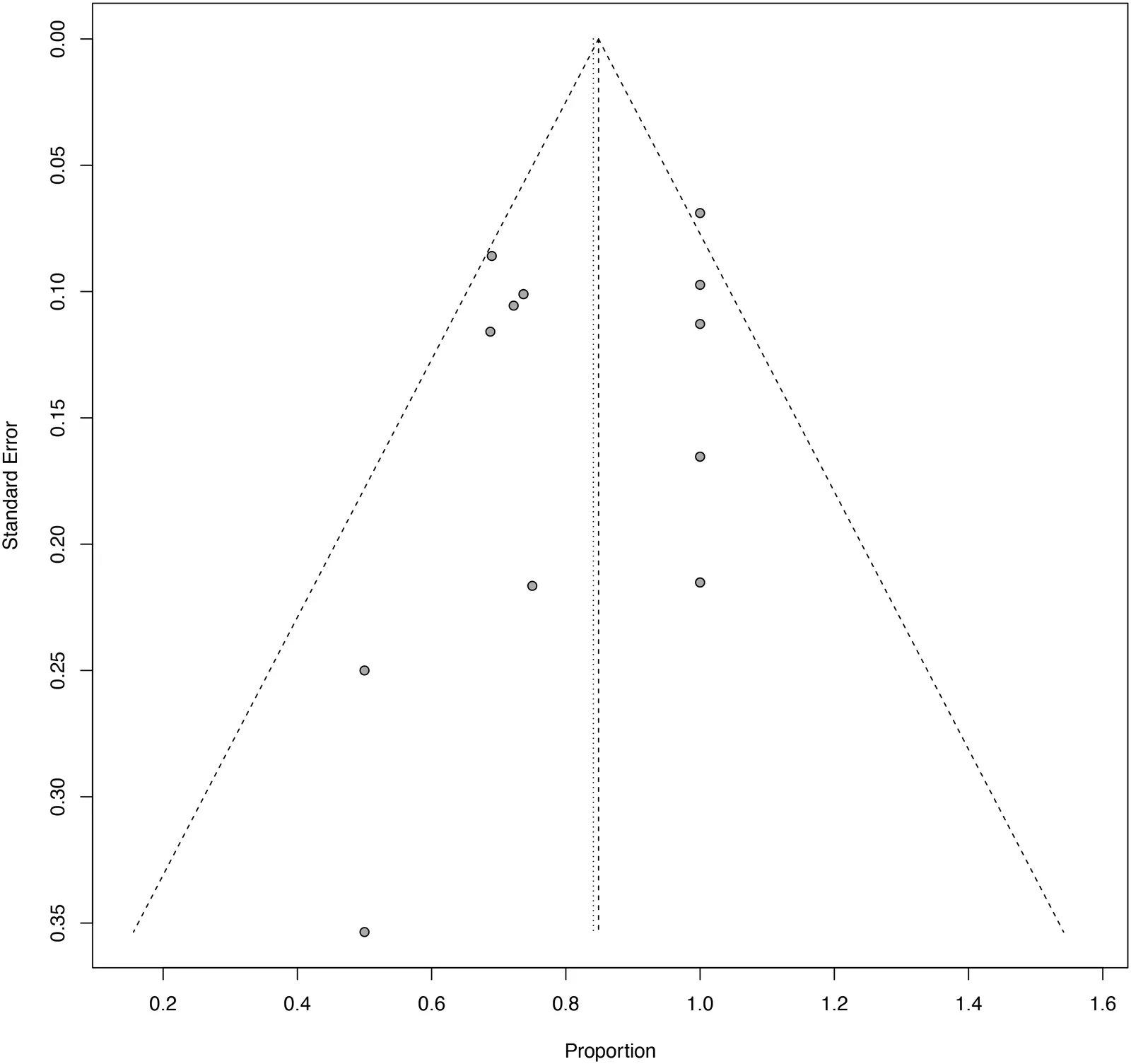
Funnel plot of overall survival after hematopoietic stem cell transplantation.

Visual inspection of the funnel plot for OS suggested some asymmetry (**Figure 6**). However, Egger’s regression test did not show statistically significant funnel-plot asymmetry (bias coefficient = −0.5292, SE = 0.9049; t = −0.58, df = 13, P = 0.569), providing no formal statistical evidence of publication bias or small-study effects for OS (**Supplementary Table 1A**). Nevertheless, publication bias could not be definitively excluded because only 15 studies were available and most were small observational cohorts or case series.

In the exploratory univariable meta-regression analyses, total study sample size was significantly and inversely associated with the reported OS proportion (β = −0.0132 per additional participant; 95% CI, −0.0204 to −0.0060; P < 0.001). NOS score was also inversely associated with reported OS (β = −0.0751 per 1-point increase; 95% CI, −0.1133 to −0.0368; P < 0.001). In both models, the estimated residual between-study heterogeneity was reduced to zero. Publication year was not a statistically significant moderator (β = −0.0081 per additional calendar year; 95% CI, −0.0184 to 0.0021; P = 0.118), with a residual I² of 32.5% and an R² of 32.2% (**Supplementary Figure 3**; **Supplementary Table 1B**). Total study sample size and NOS score were correlated (Pearson r = 0.753), indicating that these associations may reflect overlapping study-level characteristics. Given the limited number of studies and the use of aggregate study-level data, the meta-regression findings should be considered exploratory and hypothesis-generating rather than independent or causal explanations of heterogeneity.

In the leave-one-out sensitivity analysis, sequential omission of individual studies yielded pooled OS estimates ranging from 81.7% to 86.0%, compared with 84.1% in the overall analysis. I² values ranged from 28.0% to 45.5%, and no individual study materially changed the direction or magnitude of the pooled result (**Supplementary Figure 4**).

## 4 Discussion

This systematic review and meta-analysis evaluated HSCT for Krabbe disease from the perspective of immune-cell-based therapy and transplantation-related risk. The pooled results showed favorable survival outcomes, with an OS of 84% and a 5-year OS of 80%, while the pooled TRM was 7%. These findings suggest that HSCT can provide clinically meaningful life prolongation in Krabbe disease, particularly in a disorder otherwise characterized by rapid neurological deterioration and early mortality. However, the relative robustness of the survival estimate should not be extended automatically to neurological, MRI, or GVHD outcomes, which were reported in far fewer patients and were characterized by wide CIs and substantial heterogeneity. As a donor-derived cellular therapy, HSCT aims to modify disease biology through hematopoietic and myeloid cell replacement, but it also introduces alloimmune toxicities, including GVHD. Therefore, its therapeutic value depends on the balance between survival benefit, neurological preservation, and immune-related transplant risk.

Natural history cohort studies consistently report a median survival of approximately 1.1–2.5 years after birth in untreated infantile Krabbe disease, with rapid neurological decline and near-universal mortality (29–31). Against this background, the survival estimates observed in the present analysis indicate a distinct survival advantage associated with HSCT. This is particularly relevant for rare diseases such as Krabbe disease, in which randomized trials are unlikely and clinical decisions often rely on small observational cohorts. By integrating fragmented clinical evidence across studies, our analysis provides a quantitative summary of the current survival benchmark for HSCT.

Preservation of neurological function is arguably the most important outcome for patients and families, yet this was the least well documented major efficacy outcome. Only four studies including 15 patients reported neurological stability, and only four studies including 32 patients reported MRI stability. Both estimates were characterized by wide CIs and substantial heterogeneity. Improved survival was not accompanied by uniformly stable neurological or radiological outcomes. Several studies have described persistent peripheral neuropathy, progressive neuroimaging abnormalities, and ongoing functional impairment after transplantation (7, 8, 17, 25, 30, 32). In our pooled analysis, MRI stability was observed in 49% of patients, whereas neurological function stability was reported in 77%. This discrepancy suggests that HSCT may alter the natural history of Krabbe disease and prolong survival, but may not fully prevent neurological progression, particularly when irreversible injury has already occurred before transplantation. The contribution of the present review is therefore a cautious synthesis of the limited evidence and identification of a major outcome-reporting gap, rather than definitive proof of neurological stabilization.

This pattern is biologically plausible. From an immunological and cellular therapy perspective, HSCT is thought to act primarily through donor-derived hematopoietic and myeloid cells that provide functional GALC through cross-correction, thereby supporting metabolic correction, reducing psychosine-mediated toxicity, and preserving myelin integrity. Beyond enzyme replacement, experimental studies suggest that GALC-expressing macrophages and bone marrow transplantation may also influence macrophage-mediated, inflammatory, and neuroimmune pathways, which may further contribute to the disease-modifying effects of HSCT (9–11, 33–36). These mechanisms support the concept that HSCT in Krabbe disease is not merely a replacement procedure, but an immune- and cell-mediated therapeutic strategy. At the same time, the delayed and incomplete correction of established central and peripheral nervous system injury may explain why neurological outcomes remain more variable than survival outcomes.

The gap between survival benefit and neurological stabilization highlights the importance of disease stage and timing of transplantation. A cohort study including 14 symptomatic and 5 asymptomatic patients showed that HSCT improved survival and neurological outcomes in late-infantile Krabbe disease, particularly when treatment was initiated presymptomatically (17). Current clinical practice guidelines recommend HSCT for selected patients with Krabbe disease who meet early-stage criteria (37), including stage 1 of the infantile Krabbe disease staging system (38). The consensus guidelines for newborn screening, diagnosis, and treatment of infantile Krabbe disease emphasize rapid referral to centers experienced in Krabbe disease and metabolic transplantation, ideally no later than the third week of life, to allow HSCT initiation within the first four weeks of life when appropriate (6). These recommendations are consistent with the biological rationale that HSCT is more likely to preserve function when performed before extensive neuroaxonal injury has occurred.

In the present study, subgroup analyses did not identify statistically significant differences in OS according to disease onset age, pre-transplant symptom status, or age at HSCT. However, these findings should be interpreted in the context of limited data availability rather than as evidence that timing is clinically unimportant. A report of infantile-onset Krabbe disease patients transplanted within the first two months of life showed that transplantation during the first four weeks was associated with better survival and functional outcomes, including ambulation and reduced need for gastrostomy tube placement, compared with transplantation during the second month of life (27). Similarly, although another study did not show significant differences in 5- or 10-year survival between infants transplanted before or after 31 days of age, earlier transplantation was associated with better mobility and communication outcomes (18). For later-onset Krabbe disease, disease progression is typically monitored over a 3–6 month period, and eligibility for HSCT is determined based on comprehensive clinical, neurological, neurophysiological, and neuroimaging assessment (37). Taken together, these findings suggest that the absence of statistically significant subgroup differences in pooled survival should not diminish the clinical importance of early identification and careful timing of HSCT.

The exploratory meta-regression analyses provide additional context for the observed variation in OS estimates. Larger studies and studies with higher NOS scores tended to report lower OS proportions, whereas publication year was not significantly associated with OS. These findings may reflect differences in patient selection, completeness of outcome ascertainment, study design, or reporting practices rather than direct effects of sample size or methodological quality on survival. Moreover, total sample size and NOS score were correlated, suggesting that the two associations may represent overlapping study-level characteristics. Given the small number of studies and the use of aggregate data, these results should be interpreted as hypothesis-generating rather than causal.

The immune-related safety profile is central to the interpretation of HSCT in Krabbe disease. The pooled TRM rate in our analysis was 7%, which appears comparable to or lower than rates reported in pediatric HSCT populations treated for other malignant and non-malignant conditions. Large pediatric cohorts have reported early TRM rates ranging from 5.8% to 14%, depending on age, donor type, conditioning regimen, and stem cell source (39–41). However, Krabbe disease is a non-malignant and often presymptomatic indication for HSCT, and many candidates are very young infants.

GVHD-related immune toxicity deserves particular attention. The pooled incidence of aGVHD was 53%, which was higher than rates reported in broader pediatric HSCT populations, where aGVHD rates range from 8.6% to 22% (39, 42, 43). This estimate was based on a small number of patients and heterogeneous reporting of GVHD grading, donor type, conditioning regimen, stem cell source, and GVHD prophylaxis. The safety findings also require differentiated interpretation. The pooled TRM of 7% was relatively consistent, whereas the aGVHD and cGVHD estimates were derived from only five studies and had wide CIs and substantial heterogeneity. Differences in donor type, stem cell source, conditioning regimen, GVHD grading, prophylaxis, and supportive care likely contributed to this variability. Even a relatively low pooled TRM and uncertain GVHD risk must be weighed carefully in a non-malignant disorder, particularly when candidates are presymptomatic or very young infants. Future studies should therefore report GVHD, infections, engraftment, chimerism, conditioning-related complications, and immune reconstitution in a standardized manner, rather than reporting survival alone.

Survival alone also does not capture patient-centered benefit. Motor impairment, visual or auditory dysfunction, feeding dependence, communication limitations, pain or spasticity, and the need for prolonged rehabilitation may substantially affect independence and family life even when survival is prolonged. However, no included study consistently reported validated health-related quality-of-life measures or other patient- or caregiver-reported outcomes in a form suitable for quantitative synthesis. Complete GALC genotypes, residual enzyme activity, psychosine levels, and genotype-specific post-HSCT outcomes were also reported inconsistently, precluding genotype–phenotype or genotype–treatment-response analyses. Truly long-term follow-up beyond 10 years was uncommon, and no outcome could be pooled specifically for this follow-up interval.

Gene therapy is one of the most important emerging strategies for Krabbe disease. Preclinical studies using viral vector-mediated GALC delivery have shown the potential to improve enzymatic activity, reduce psychosine-related toxicity, slow demyelination, and prolong survival (12–14). AAV-based gene therapy, intrathecal gene delivery, and combined gene-cell approaches are therefore being investigated as potential ways to achieve more direct or sustained metabolic correction within the nervous system (44). Compared with conventional HSCT, these approaches may theoretically reduce some transplant-related immune toxicities and improve central nervous system targeting. However, most evidence remains preclinical or early translational, and long-term clinical data on neurological function, MRI progression, safety, durability, and quality of life are still limited.

Therefore, HSCT should not be viewed as the final therapeutic endpoint for Krabbe disease, but rather as the current clinical benchmark against which emerging therapies can be compared. The pooled estimates of OS, 5-year OS, TRM, GVHD, MRI stability, and neurological stability reported here may help future studies evaluate whether gene therapy, modified cellular therapy, or combined approaches can complement or surpass current HSCT-based strategies, particularly with respect to meaningful neurological preservation.

This study has several strengths. It was conducted according to PRISMA 2020 guidance, was prospectively registered in PROSPERO, and integrated both efficacy and immune-related safety outcomes across rare-disease transplant cohorts. By combining survival, neurological, imaging, TRM, and GVHD outcomes, this review provides a structured evidence base for individualized counseling, transplant referral, and future trial design.

Several limitations should also be acknowledged. First, because Krabbe disease is exceedingly rare, the available evidence was derived mainly from small, non-randomized observational studies, which limits the precision of pooled estimates. Although Egger’s regression test did not detect statistically significant funnel-plot asymmetry, publication bias or small-study effects cannot be completely excluded. Second, neurological, MRI, and GVHD outcomes were reported in relatively few studies and were assessed using heterogeneous definitions, resulting in wide confidence intervals and limited generalizability. Third, inconsistently reported transplant characteristics and long-term patient-centered outcomes restricted more detailed subgroup and meta-regression analyses. Nevertheless, by systematically integrating the available survival, functional, radiological, and safety data, this review provides a useful summary of the current evidence and identifies key priorities for future prospective, standardized, and international studies.

## 5 Conclusion

HSCT is associated with favorable survival and relatively low pooled TRM in selected patients with Krabbe disease, and the pooled OS estimate remained robust in leave-one-out analysis. However, survival should not be equated with preservation of neurological function. Evidence for neurological stability, MRI stability, and GVHD remains limited, heterogeneous, and imprecise because these outcomes were reported in few studies and patients. Nevertheless, by integrating these outcomes and distinguishing relatively well-supported survival estimates from key functional evidence gaps, this study provides clinically useful benchmarks for risk–benefit counseling and future therapeutic development. Future prospective studies and registries should prioritize standardized long-term neurological, MRI, and quality-of-life outcomes.

## Data Availability

The original contributions presented in the study are included in the article/**Supplementary Material**. Further inquiries can be directed to the corresponding author.

## Supplementary material

Supplementary Figure 1 Complete literature search strategy.

Supplementary Figure 2 Transplant age and follow-up duration. **(A)** Age at HSCT. **(B)** Follow-up duration after HSCT.

Supplementary Figure 3 Exploratory univariable meta-regression analyses of overall survival after hematopoietic stem cell transplantation. Bubble plots show the associations of reported OS with **(A)** total study sample size, **(B)** publication year, and **(C)** Newcastle–Ottawa Scale score. Bubble size represents the relative inverse-variance weight of each study. Solid lines represent the fitted meta-regression relationships, and shaded areas represent the corresponding 95% confidence intervals.

Supplementary Figure 4 Leave-one-out sensitivity analysis of overall survival after hematopoietic stem cell transplantation. Each row presents the pooled random-effects OS estimate after sequential omission of the indicated study. The pooled estimates ranged from 81.7% to 86.0%, indicating that no single study materially influenced the overall result.

Supplementary Table 1 Assessment of funnel-plot asymmetry and exploratory meta-regression analyses for overall survival after hematopoietic stem cell transplantation.
