# Supplementary material for: Survival benefit and immune-related toxicities after hematopoietic stem cell transplantation in Krabbe disease: a systematic review and meta-analysis
Source: Front Immunol. 2026 Aug 13;17:1924827. doi: 10.3389/fimmu.2026.1924827 (PMC13527937; doi:10.3389/fimmu.2026.1924827)
Supplement: Supplementary file 1 [file Table1.docx]

**Supplementary Table S1. Assessment of funnel-plot asymmetry and exploratory meta-regression analyses for overall survival after hematopoietic stem cell transplantation**

**Panel A. Egger's regression test for funnel-plot asymmetry**

| **Outcome** | **Studies, k** | **Test** | **Bias coefficient (SE)** | **t (df)** | **P value** | **Interpretation** |
| --- | --- | --- | --- | --- | --- | --- |
| Overall survival | 15 | Egger's linear regression test | -0.5292 (0.9049) | -0.58 (13) | 0.5687 | No statistically significant funnel-plot asymmetry |

**Panel B. Exploratory univariable meta-regression analyses**

| **Moderator** | **Studies, k** | **Unit** | **β (95% CI)** | **P value** | **Residual I²** | **R²** |
| --- | --- | --- | --- | --- | --- | --- |
| Total sample size | 15 | Per additional participant | -0.0132 (-0.0204 to -0.0060) | <0.001 | 0% | 100% |
| Publication year | 15 | Per additional calendar year | -0.0081 (-0.0184 to 0.0021) | 0.118 | 32.5% | 32.2% |
| NOS score | 15 | Per 1-point increase | -0.0751 (-0.1133 to -0.0368) | <0.001 | 0% | 100% |

**Abbreviations:** CI, confidence interval; HSCT, hematopoietic stem cell transplantation; NOS, Newcastle-Ottawa Scale; OS, overall survival; SE, standard error. **Notes:** Egger's linear regression test was performed for the primary outcome of OS. A two-sided P value <0.05 was considered indicative of statistically significant funnel-plot asymmetry; a non-significant result does not definitively exclude publication bias or small-study effects. Separate exploratory univariable mixed-effects meta-regression models were fitted for total study sample size, publication year, and NOS score. Untransformed proportions were analyzed using the inverse-variance method, with between-study variance estimated using the DerSimonian-Laird method. β represents the absolute change in the reported OS proportion associated with a one-unit increase in the moderator. Negative coefficients indicate lower reported OS estimates with increasing moderator values. Residual I² represents the proportion of variability remaining after inclusion of the moderator, and R² represents the proportional reduction in estimated between-study variance. Total sample size and NOS score were correlated (Pearson r = 0.753); therefore, their associations may reflect overlapping study-level characteristics and should not be interpreted as independent or causal effects. Because only 15 studies were available and aggregate study-level data were used, all meta-regression findings should be considered exploratory and hypothesis-generating.
